# Decorin knockdown affects the gene expression profile of adhesion, growth and extracellular matrix metabolism in C-28/I2 chondrocytes

**DOI:** 10.1371/journal.pone.0232321

**Published:** 2020-04-30

**Authors:** Mengying Wang, Zhengzheng Li, Meng Zhang, Hui Wang, Ying Zhang, Yiping Feng, Yinan Liu, Jinghong Chen

**Affiliations:** Institute of Endemic Diseases, School of Public Health, Xi’an Jiaotong University Health Science Center, Xi’an, Shaanxi, PR China; University of Crete, GREECE

## Abstract

Decorin is a member of small leucine-rich proteoglycan family, which is involved in multiple biological functions mainly as a structural and signaling molecule, and disturbances in its own metabolism plays a crucial role in the pathogenesis of osteoarthropathy. In this study, we aim to further explore the biological function of decorin and their role in human chondrocyte cell line, C28/I2. Lentivirus-mediated shRNA was applied to down-regulate decorin expression in C28/I2 chondrocytes. Effect of decorin knockdown on gene expression profiles was determined by RNA sequencing followed by bioinformatics analysis. MTT, adhesion assays and flow cytometry were used to investigate the effect of decorin knockdown on cell proliferation, adhesion, and apoptosis. sGAG content in the culture medium was determined by DMMB assay. Stably transfected C28/I2 cells were seeded onto the cancellous bone matrix gelatin (BMG) to construct tissue-engineered cartilage. The histological patterns were evaluated by H&E and Toluidine blue staining. In this study, 1780 differentially expressed genes (DEGs) including 864 up-regulated and 916 down-regulated genes were identified using RNA-Seq. The reliability of the gene expression was further verified by qRT-PCR. GO and KEGG pathway enrichment analysis revealed diverse cellular processes were affected by decorin silencing such as: cell adhesion, growth, and metabolism of extracellular matrix. In addition, we confirmed that down-regulation of decorin significantly suppressed cell proliferation and adhesion and induced apoptosis. The sGAG content in the media was significantly increased after decorin silencing. Engineered articular tissues in the decorin knockdown group exhibited cartilage destruction and proteoglycan loss as evidenced by H&E and Toluidine blue stains. Overall, this combined data helps to provide a comprehensive understanding of the roles of decorin following its knockdown in C28/I2 cells.

## Introduction

Decorin is a prominent member of the class I small leucine-rich proteoglycan (SLRP) family in the extracellular matrix (ECM), and consists of a core protein and a single chondroitin/dermatan sulfate glycosaminoglycan (GAG) side chain at the N-terminal. Decorin was initially defined as structural components modulating the synthesis, organization, and assembly of collagen fibrils [1,2], but with time has evolved as a versatile proteoglycan, involved in various biological processes such as cell proliferation, differentiation [3], matrix adhesion [4], autophagy [5], inflammation and immunity [6], angiogenesis [7], tumorigenesis [8], osteoarthritis and osteoporosis [9–11], and fibrosis [12].

Several literature sources demonstrate that altered decorin is involved in the pathogenesis of many diseases [9,11,13,14]. Decorin is highly expressed in cartilage, is a key matrix constituent for the extracellular matrix and is involved in proper cartilage biomechanical functions. Within the hyaline cartilage tissue, decorin is present in pericellular and territorial, as well as in interterritorial matrix of cartilage [4,15,16]. In osteoarthritis (OA), the small proteoglycans (PG), decorin and biglycan, are lost from the surface of articular cartilage; however, their contents increase in the deeper parts of the cartilage [17]. Extensive degradation of both large and small PGs, such as decorin and biglycan were found in cartilages of patients suffering from OA and rheumatoid arthritis (RA) [9,11,18]. Additionally, the importance of decorin in cartilage biology was highlighted by the phenotype of decorin knockout mice, which showed that the depletion of decorin and biglycan induced progressive loss of bone mass, causing OA and an osteoporosis-like phenotype [19]. Recent studies have reported that decorin deficiency leads to the altered ECM biomechanical properties and cartilage stiffness, and decorin-null mice are less prone to develop OA after forced exercise [20].

Although degradation of decorin might disturb the homeostasis of cartilage tissues, the comprehensive molecular mechanisms underlying decorin depletion have not yet been elucidated. The present study aimed to identify the differences in gene expression and underlying molecular changes modulated by decorin in a chondrocyte cell line, C28/I2. Effect of decorin silencing on gene expression profiles was analyzed by RNA sequencing (RNA-seq). By using integrated bioinformatics analysis, we further identified the differentially expressed genes (DEGs) and pathways regulated by decorin, which could improve our understanding of the underlying molecular events regulated by these genes in osteoarticular diseases.

## Materials and methods

### Cell culture

Human chondrocyte cell line C28/I2, was kindly provided by Professor Mary B Goldring (Hospital for Special Surgery, Weill Cornell Medical College, New York, USA). The cells were cultured in DMEM/F12 supplemented with 10% FBS and 1% Penicillin/Streptomycin in a humidified 5% CO_2_ incubator at 37°C. HEK293T packaging cells for production of lentiviruses were cultivated in DMEM high glucose, with 10% FBS in a 5% CO_2_ atmosphere at 37°C.

### Lentivirus packaging and cell transfection

The DCN Human shRNA Plasmid Kit (Cat. No. TL313548) and lentiviral packaging kits (Cat. No. TR30037) were purchased from Origene Technologies Inc (OriGene, Rockville, MD, USA). For production of lentiviruses, pGFP-C-shLenti plasmids coding for decorin-specific shRNAs and the packaging plasmid pCMV 8.91(1:1.2 ratio) were co-transfected into HEK293T cells using MegaTran 1.0 transfection reagent (Cat. No. TT200002, OriGene, Rockville, MD, USA). The DNA-lipid complex was removed after overnight incubation, and fresh medium was added to the cells. At 48 h and 72 h post transfection, the supernatant containing the virus was collected and filtered through a 0.45μm PVDF membrane (Millipore). The targeted C28/I2 cells were infected by incubating with lentivirus for 24 h. Stable cell lines were obtained by cultured in medium containing puromycin for 2 weeks, and the selected cells were prepared for the subsequent experiment.

### RNA sequencing and bioinformatics analysis

Total RNA was extracted using Trizol reagent (Invitrogen, Carlsbad, CA, USA) according to the manufacturer’s protocol. For strand-specific library construction and sequencing, residual rRNAs were removed from the total RNA, which were then fragmented and reverse transcribed into cDNA with random primers. Second-strand cDNA were synthesized by mixing dNTPs, DNA polymerase I, RNase H, and buffer. The cDNA fragments were purified using a QiaQuick PCR extraction kit (Qiagen). Next, the second-strand cDNAs were end-repaired, poly (A) tails added, and were ligated to Illumina sequencing adapters. The ligation products were size-selected by agarose gel electrophoresis, amplified by PCR and sequenced using the HiSeq 2500 sequencer (Illumina). For bioinformatics analysis, raw reads containing adapters of low quality (Q-value≤ 20) were removed, and then mapped to the human reference genome (GRCh38.p12) by TopHat2 (version2.0.3.12). For differential expression analysis, gene abundances were quantified by edgeR package, and DEGs were selected based on a false discovery rate (FDR) <0.05 and |log_2_ fold change (FC)| >1. We used the Gene Ontology Consortium (http://www.geneontology.org/) system and Kyoto Encyclopedia of Genes and Genomes (KEGG, http://www.genome.jp/kegg/.html) database for categorizing the identified genes and identifying relevant biological pathways of clustered DEGs.

### Cell proliferation assay

For MTT assay, C28/I2 cells transfected with the specific or control shRNA were seeded on 96-well plates (2,000 cells/well) and incubated for 24 h. 20μl of MTT (5 mg/mL) solution was added to the medium and incubated for an additional 4 h. The formazan crystals in each well were solubilized in DMSO, and the absorbance was measured at a wavelength of 490 nm on a microplate reader.

### Cell adhesion assay

Cell adhesion assays were performed in 96-well plates coated with fibronectin (FN, Cat. No. F1141, Sigma- Aldrich, St. Louis, MO, USA) and blocked with 1% bovine serum albumin (BSA; CAS. No. 9048-46-8, Sigma- Aldrich, St. Louis, MO, USA) for 1 h at 37°C before seeding the C28/I2 cells. After removing the excess fibronectin solution from the wells, 2 × 10^3^ cells suspended in 100μL medium were seeded in each well and incubated for 2 h. The medium was subsequently discarded and the adhering cells were fixed with 4% paraformaldehyde and then stained with 0.5% crystal violet (Sangon Biotech, Shanghai, China) for 10 min; the cells were photographed using a phase-contrast microscope. After washing, the dye is eluted with 10% acetic acid (Sigma- Aldrich, St. Louis, MO, USA) and absorbance was measured at 540 nm.

### Flow cytometry analysis

Cell apoptosis rate was analyzed using the PE AnnexinV Apoptosis Detection Kit (Cat. No. 559763, BD Biosciences, San Diego, CA, USA). C28/I2 chondrocytes were collected and washed twice with ice-cold PBS. Cells were double-stained with PE Annexin V and 7-Amino-Actinomycin (7-AAD) and then analyzed using the BD FACSCalibur^™^ flow cytometer.

### Dimethylmethylene blue (DMMB) assay

Sulfated glycosaminoglycan (sGAG) release into the conditioned medium was determined by 1, 9-dimethyl-methylene blue assay (Cat. No. 341088, Sigma-Aldrich St. Louis, MO, USA). Cells were seeded at a same density in 6-well plates and incubated for 3 days. The culture medium of each sample was collected, forty microliters of the supernatant of each sample and chondroitin sulfate (CS) standards were transferred to a 96-well microplate and mix with 200 μl of the DMMB dye. Absorbance was measured at 525 nm within 10 min using a microplate reader. sGAGs concentration were calculated based on the OD values and normalized to the standard curve.

### Quantitative reverse transcription PCR (qRT-PCR)

Total RNA was extracted from C28/I2 chondrocytes using TRIzol reagent. One microgram RNA was reverse transcribed to cDNA using the RevertAidTM First-Strand Synthesis kit (Fermentas, Waltham, MA, USA). qRT- PCR analysis was carried out using Fast-Start Essential DNA Green Master Mix (Roche, Germany) on an Applied Biosystems 7000 (ABI). The thermal cycling conditions for all reactions were as follows: activation10 min at 95°C, followed by 40 amplification cycles of 95°C for 10 sec, 60°C for 10 sec and 72°C for 10 sec. Gene expression was calculated according to the 2^-ΔΔCt^ comparative method. The sequences of primers are listed in Table 1.

**Table 1:** Primer sequences for reverse transcription-qPCR.

Gene	Primer sequence (5′-3′) Forward:	Reverse:	Size (bp)
**Decorin**	5'- CCTGGACACAACACCAAAAAGG -3'	5'- ATCTGAAGGTGGATGGCTGTATCT -3'	94
**ICAM-3**	5’-CTTAACCGCTGTGCTTCCGT -3’	5’- CCATGGTGGCCATTCTGACA -3’	105
**IL-6**	5'- AAGCCAGAGCTGTGCAGATGAGTA -3’	5'- TGTCCTGCAGCCACTGGTTC -3′	150
**Cdh6**	5'- AAGTTCTCGACGTCAACGACAATG -3′	5'- ACAGCACGCAGGGTCTGAATC -3′	106
**Cdh11**	5'- GGTCTGGAACCAGTTCTTCG -3'	5′- TCTCGATCCAACGTCTTGGT -3′	192
**BMP-5**	5'- CGCATACAGTTATCTCG -3’	5'- CTTTGTAATGCCTTCG -3′	160
**GAPDH**	5'- CAAAGTTGTCATGGATGACC -3'	5'- CCATGGAGAAGGCTGGGG -3'	195

### Western blot analysis

Proteins were extracted from each well using RIPA lysis buffer, BCA^™^ Protein Assay kit (Pierce, Appleton, WI, USA) was used to determine protein concentration. Equal amounts of proteins were subjected to 10% SDS-PAGE gel and then transferred onto PVDF membranes (Merck Millipore, Billerica, MA, USA). The membranes were blocked in 5% non-fat milk and then incubated overnight at 4°C with primary antibodies against decorin (1:400, Proteintech, USA) or GAPDH (1:1000, Proteintech, USA). Subsequently, the membranes were washed with TBS-T and incubated for 1 h at room temperature with peroxidase-conjugated secondary antibodies. Protein bands were detected using an enhanced chemiluminescence detection system (Thermo Scientific) and visualized with the Bio-Rad ChemiDoc^™^ XRS system.

### Tissue engineered cartilage preparation and histology

Demineralized bone matrix gelatine (BMG) was prepared and harvested from freshly euthanized adult Japanese white rabbits (from the Experimental Animal Centre of Xi’an Jiaotong University) as described previously [21,22]. All protocols and procedures were approved by Institutional Animal Care and Use Committee of Xi’an Jiaotong University in June 2015 (Permission no. XJTULAC201-249). Rabbits were anesthetized with isoflurane, and all efforts were made to minimize suffering. Prior to using, BMG scaffolds were sterilized with ethylene oxide for 48 h and then degassed for further evaluations. The prepared BMG scaffolds were pelleted down at chambers (8 μm pore size, Costar 3422; Corning, NY, USA), which were placed in 24-well plates. Stable transfection C28/I2 cells (1.0×10^6^) resuspended in 100μl medium were seeded on BMG scaffolds for 24 h. After four hours, 3 ml culture medium containing 10% FBS was carefully added to the chondrocytes-cancellous BMG construct, and continuously cultured for 21 days. The medium was changed every 2 days.

For histology, the engineered cartilage was fixed in 4% (w/v) paraformaldehyde, dehydrated in a gradient ethanol and xylene, and then embedded in paraffin. Serial 6-μm-thick sections were prepared using a microtome. After deparaffinization, sections were stained with 0.04% Toluidine blue solution for 5 min and differentiated with 0.1% acetic acid for 10–20 sec. Sections were washed with tap water for 5 min, dehydrated with increasing concentrations of ethanol, cleared in xylene for 3 min, and mounted with resinous mounting media. Each specimen was also stained with Mayer's Hematoxylin for 6 min, rinse briefly in tap water, blue in the Scott's tap water for 2 min. Following flushing with tap water for 2 min, sections were stained with Eosin for 1 min and then washed with running water. Finally, sections were dehydrated through 95% alcohol and 2 changes of absolute alcohol, cleared in xylene, and then mounted with resinous mounting medium.

### Statistical analysis

All the data are shown as mean ± standard deviation (SD). Student's t test was utilized for comparison of two groups, multiple comparisons were analyzed by variance (ANOVA) with either Dunnett's or Tukey’s post-test. *P* values less than 0.05 was considered statistically significant.

## Results

### Silencing of decorin expression in C28/I2 cells

**Fig 1:**
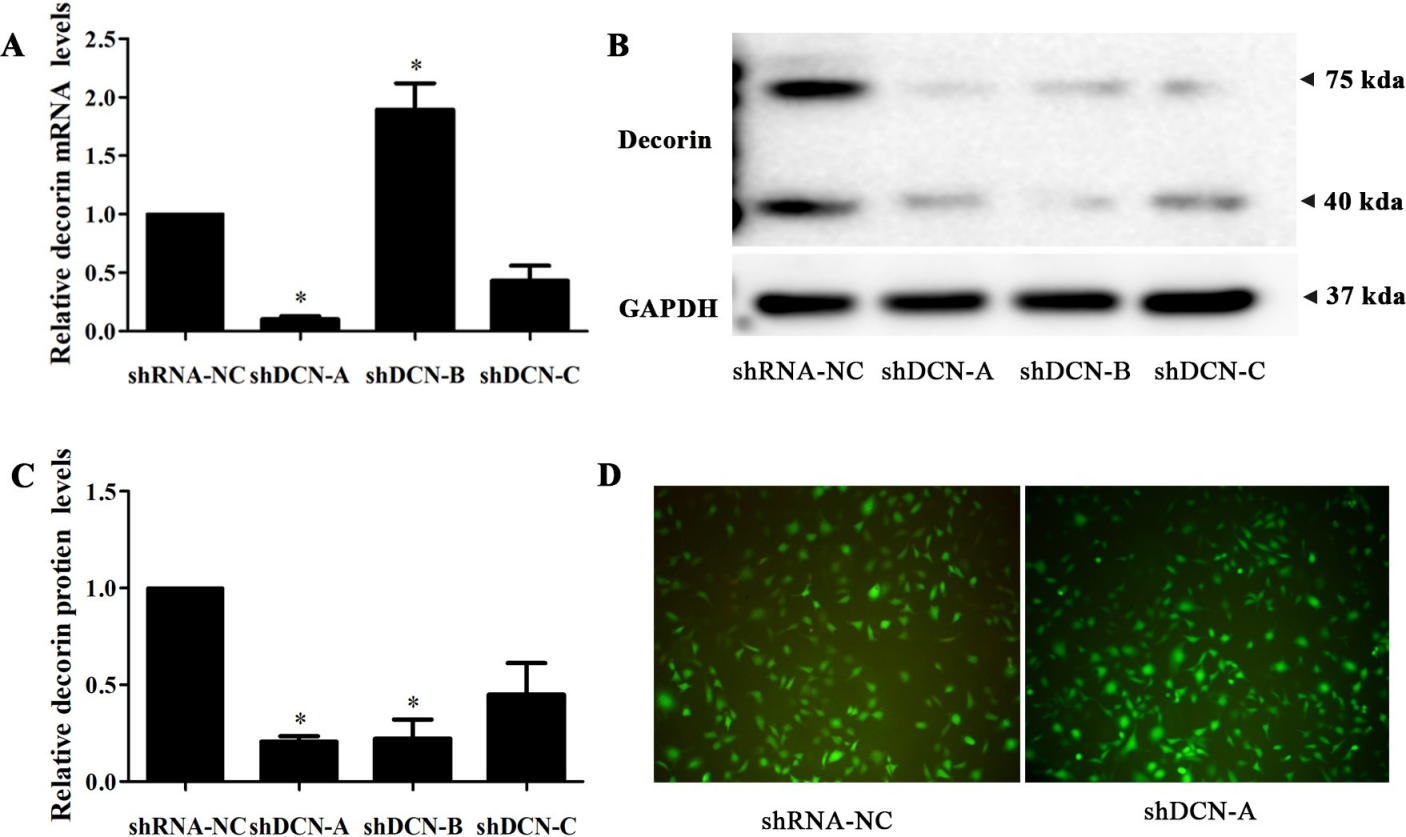
The silencing efficiency of decorin knockdown in C28/I2 cells. C28/I2 cells were transfected with decorin-shRNA, the knockdown efficiency was verified by quantitative RT-PCR (A) and western blot analysis (B, a representative image, C, statistical analysis). Transfection efficiency was visualized by fluorescence microscopy (D). GAPDH was used as a reference gene. Data are shown as mean ± SD from three independent experiments, *P < 0.05, compared with the shRNA-NC cells.

We employed lentivirus-mediated shRNA for stable knockdown of decorin expression in human C28/I2 cell lines. Three shRNA constructs targeting decorin and one empty vector were separately transfected into C28/I2 cells, and their silencing efficiency was detected by both qRT-PCR and Western Blot. After transfection, decorin mRNA and protein expression were significantly reduced in shRNA-DCN-A group when compared with those in shRNA-NC group (Fig 1A–1C). Number A sequence pairs were used in the subsequent study. The transfection efficiency was detected by observing the fluorescence efficiency (more than 90%) under a fluorescence microscope (Fig 1D).

### Distinct gene expression profile after decorin knockdown and qRT-PCR verification in C28/I2 cell

**Fig 2:**
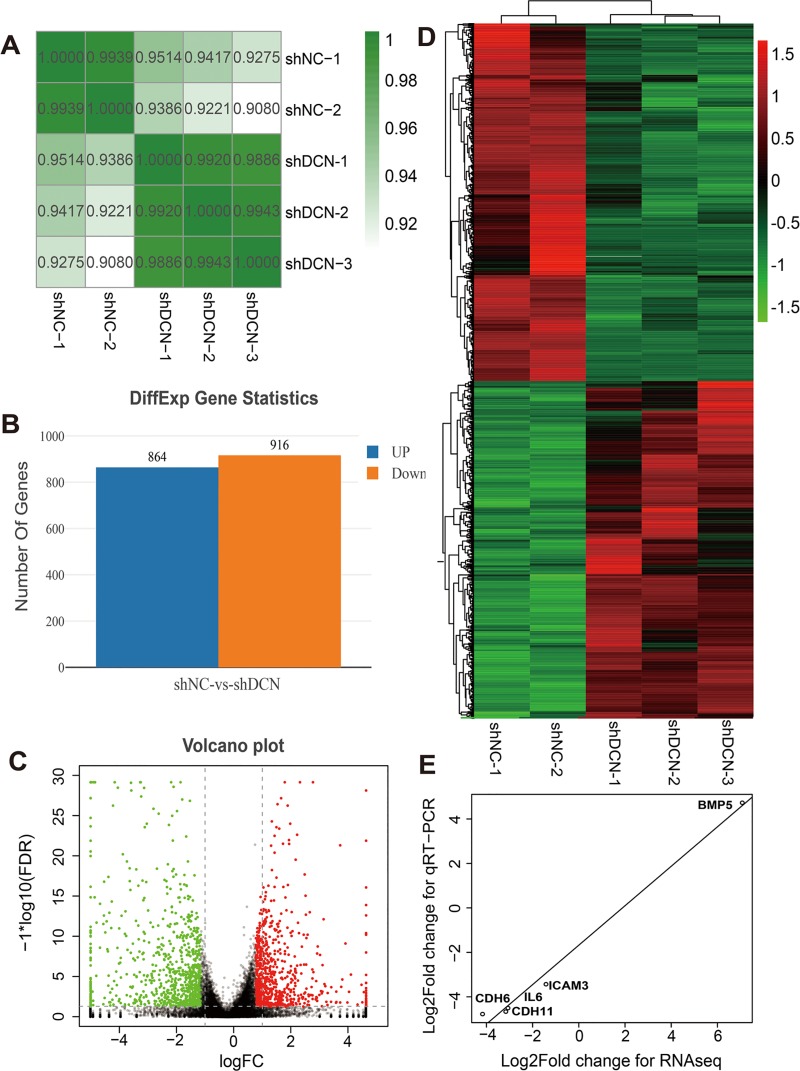
Result of RNA-Seq data analysis. (A) Pearson’s correlation analysis of normalized expression levels of the samples with average linkage and the respective values are given in the table aside. The greater the absolute value of r, the stronger the correlation. (B) Statistical analysis of differentially expressed gene numbers. (C) Distribution of the DEGs shown as a volcano plot. |log_2_ FC| >1 and FDR < 0.05 were set as a cut off. (D) Hierarchical clustering map of gene expression profiles of C28/I2 cells. Different colors indicate the relative fold expression of each gene: red points represent up-regulated genes, green points indicate genes down-regulated, and black points indicate no change in expression levels. (E) Validation of the expression data from RNA-Seq assay by qRT-PCR. Scatter plots represent log_2_ transformed fold change values of a single gene from qRT-PCR (X-axis) and RNA-Seq analysis (Y-axis).

To better understand the implications of decorin silencing in C28/I2 cell lines, RNA-Seq was performed to examine differentially expressed genes before and after decorin knockdown. The correlation between gene expression levels in different samples indicated biological repetition (Fig 2A). After decorin knockdown, a total of 1780 genes were differentially expressed in decorin-knockdown group compared with shRNA-NC group (S1 Table). Among them, 864 genes (48.53%) were upregulated, while 916 genes (51.46%) were downregulated (FDR < 0.05 and |log_2_ FC| >1) (Fig 2B). The distribution of the differentially expressed genes is shown in a volcano plot (Fig 2C). Hierarchical clustering was used to analyze the changes in expression patterns. There was a significant difference in expression patterns of genes between the shRNA-DCN and shRNA-NC cells (Fig 2D).

To verify the reliability of RNA-Seq data, 3 genes closely related to cell adhesion and 2 random genes were selected for qRT-PCR analysis and the expression profiles of the candidate unigenes by qRT-PCR were consistent with the RNA-Seq data, suggesting a reliable transcriptome analysis using RNA-Seq (Fig 2E).

### Functional enrichment analysis of differentially expressed genes

**Fig 3:**
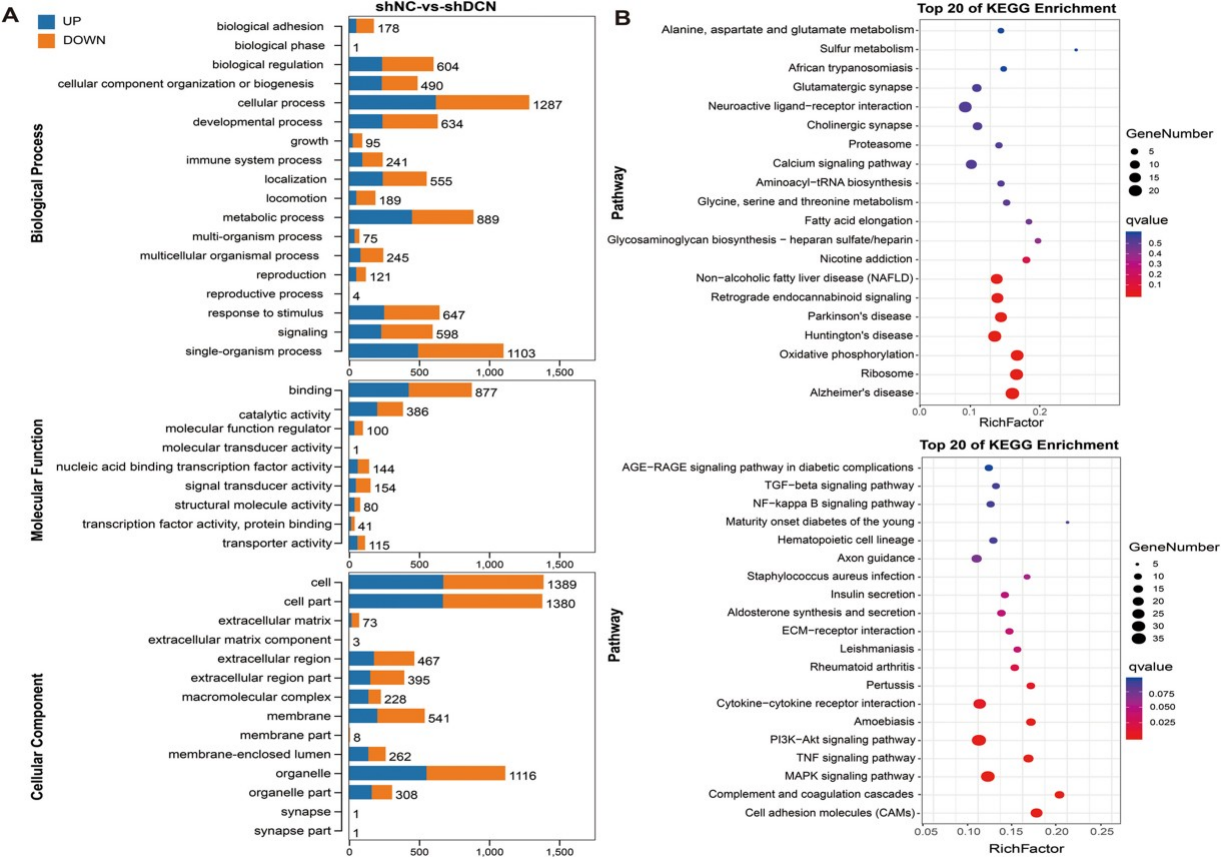
GO and KEGG pathway analyses were conducted for DEGs. (A) Histogram of GO analysis of up- and down-regulated genes upon decorin knockdown. (B) Top 20 pathways of up- and down-regulated genes in the KEGG pathway analysis. Rich Factor refers to the ratio between the number of DEGs and the number of GO annotations in this pathway. The color of circles denotes the range of the *P* value, and the circle's scale indicates the gene ratio. The larger the circle and the lower the *P* value, the more enriched and meaningful the pathway.

In order to gain an overall insight into the functions of the annotated genes, the GO functional classification was performed. The DEGs were categorized into three major functional types: Biological Process (BP), Cellular Component (CC), and Molecular Function (MF). As shown in Fig 3A, the significantly enriched GO terms were mainly distributed in the BP and CC categories. DEGs in the BP category were significantly enriched in biological adhesion, cellular process, metabolic process, biological regulation, developmental process, and growth; whereas those in CC category was enriched in cell part, extracellular region, extracellular region part, extracellular matrix, and extracellular matrix component. Besides, DEGs in the MF category were significantly enriched in binding, catalytic, and signal transducer activity.

The results of KEGG pathway enrichment analysis are summarized in Fig 3B. The diagram indicates that the downregulated genes were mostly enriched in cell adhesion molecules (CAMs), complement and coagulation cascades, Mitogen-activated protein kinases (MAPKs) signaling pathway, Tumor Necrosis Factor (TNF) signaling pathway, PI3K-Akt signaling pathway, cytokine-cytokine receptor interaction, rheumatoid arthritis, ECM-receptor interaction, insulin secretion, NF-kappa B signaling pathway, etc. Also, the elevated expressions of genes were likely to be involved in ribosome, oxidative phosphorylation, glycosaminoglycan biosynthesis-heparan sulfate/ heparin, glycine, serine and threonine metabolism, sulfur metabolism, and some nervous system disease-related pathways. Both the GO and KEGG pathway analysis show that decorin was involved in the regulation of cell adhesion, growth, and ECM degradation by some signaling pathways.

### Knockdown of decorin suppresses cell proliferation, adhesion, and induces apoptosis of C28/I2 cells

**Fig 4:**
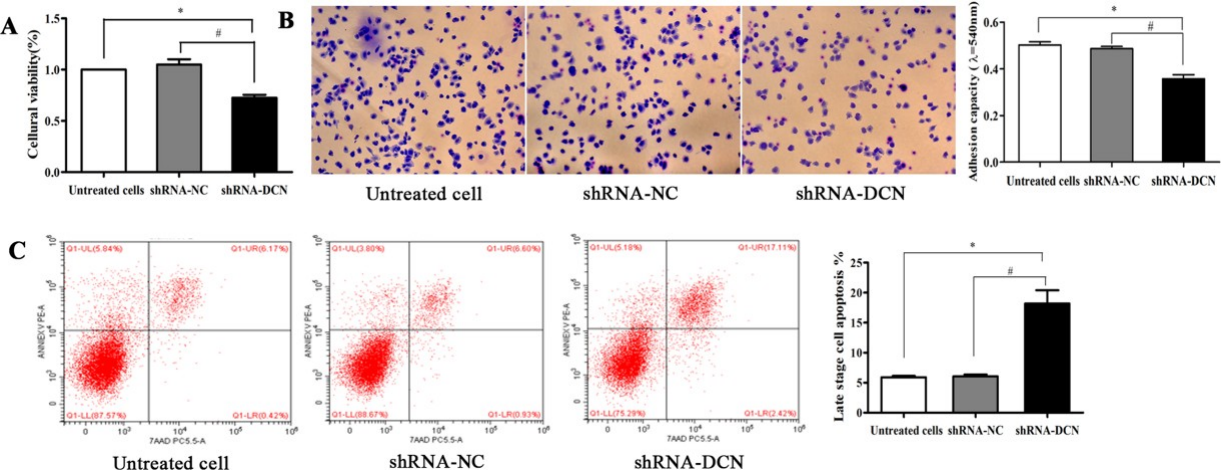
Effects of decorin knockdown on cell proliferation, adhesion, and the apoptosis of C28/I2 cells. Decorin knockdown inhibited C28/I2 cell proliferation, as detected by the MTT assay (A). Cell adhesion assay showed that decorin knockdown induced a remarkable reduction of the cell adhesion ability of these cells (B). Cell apoptosis was measured by PE Annexin V/7-AAD staining (C). The results are shown as mean± SD for triplicate determinations. Magnification, ×200. *P < 0.05, compared with untreated cells. ^*#*^*P* < 0.05, compared with shRNA-NC cells.

To determine the impact of the proteoglycan decorin on C28/I2 cell proliferation, MTT assay was conducted. Cell proliferation was significantly suppressed in decorin- knockdown group compared with that in the control group (untreated group and shRNA-NC group) (Fig 4A). Moreover, the adhesion assay showed that knockdown of decorin significantly suppressed adhesion ability of C28/I2 cells (*P* < 0.05) (Fig 4B). Additionally, PE Annexin V/7-AAD staining was conducted to evaluate cell apoptosis, and the results demonstrated that silencing of decorin remarkably elevated late stage apoptotic rates from (5.89 ± 0.25)% in untreated cells and (6.03 ± 0.32)% in shRNA-NC group to (18.18 ± 2.22)% in shRNA-DCN group (*P* < 0.05) (Fig 4C).

### Effect of decorin knockdown on sGAG

**Fig 5:**
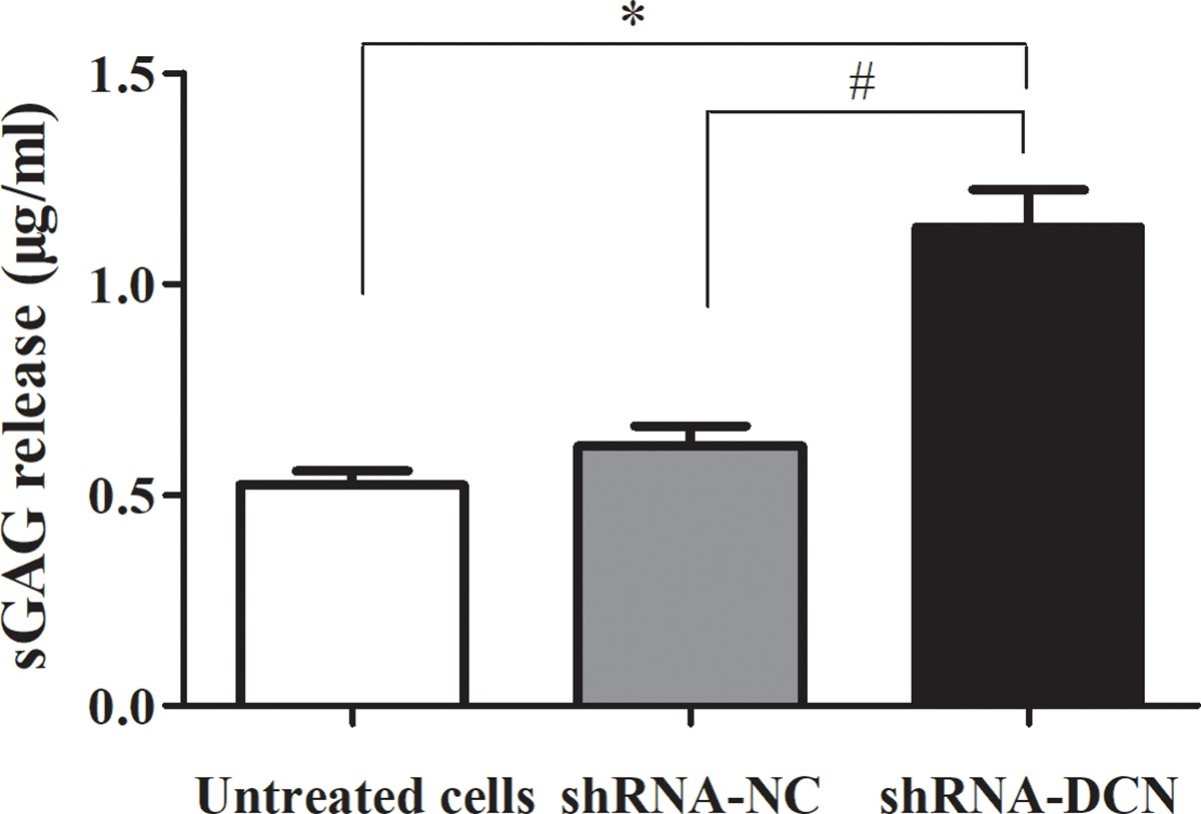
Effects of decorin knockdown on sGAG content. DMMB assay was performed to quantify sGAG content in conditioned medium of C28/I2 cells after decorin-shRNA transfection. Results are shown as means ± SD of three independent experiments. *p < 0.05 compared with untreated cells; #*p* < 0.05 compared with shRNA-NC cells.

We also measured the content of sulfated glycosaminoglycan (sGAG) secreted by chondrocytes in culture. As shown in Fig 5, the sGAG release were significantly increased in shRNA-DCN group when compared with that in control group (untreated group and shRNA-NC group).

### Histological changes of the tissue engineered cartilage after decorin knockdown

**Fig 6:**
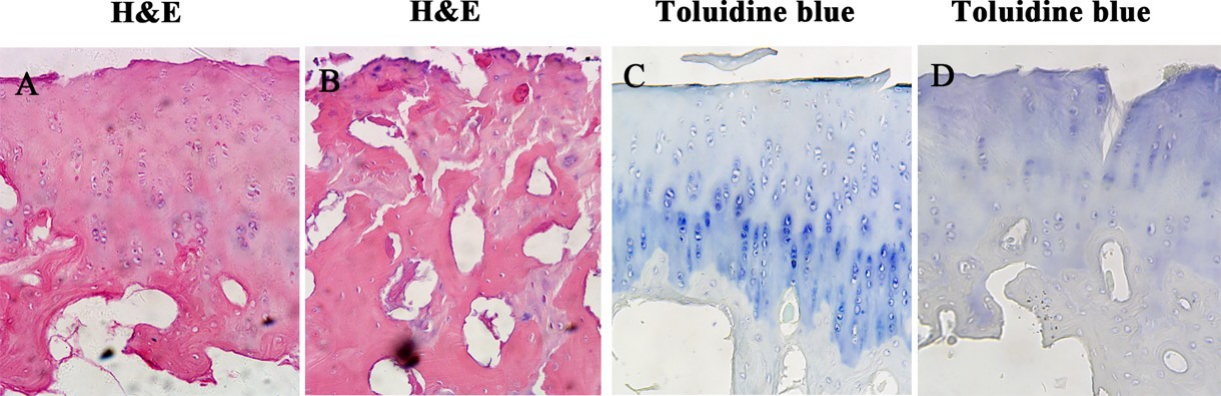
Histological analysis of the engineered cartilage tissue after decorin knockdown. Representative images of engineered-tissue cartilage from shRNA-NC and shRNA-DCN group stained with H&E (A, B); Representative images of engineered-tissue cartilage from shRNA-NC and shRNA-DCN group stained with Toluidine blue (C, D).

The morphology of tissue-engineered cartilage was evaluated by H&E staining, we found that the shRNA-NC group had a regular cell arrangement and homogeneous staining (Fig 6A). In contrast, DCN knockdown triggered slight cartilage destruction, with disordered arrangement of chondrocytes, and loss of alkalinity in the ground substance (Fig 6B). Toluidine blue stained sections showed a reduction of the staining and loss of proteoglycans in the decorin knockdown tissue cartilage (Fig 6C and 6D).

## Discussion

Changes in SLRPs gene expression occur in many disease models and patients, including cancer [6, 13] skin diseases [23], and arthritis [9–11, 17–20]. In OA, a loss of the proteoglycans, aggrecan and decorin, occur at the surface of articular cartilage [17, 24]. At late stages of OA, the mRNA and protein levels of decorin and biglycan are up-regulated, probably as an attempt to repair damaged ECM, characteristic of this stage of the disease [25]. Breakdown of the synthesis and degradation of cartilage matrix components is a common feature of both RA and OA. The SLRPs family members are suggested to be crucial for maintaining normal chondrocyte activity and cartilage homeostasis, and alterations in the expression and distribution of SLRPs could be involved in the progression of osteoarthropathy. In this study, we performed RNA-Seq analysis to examine gene expression changes in response to decorin knockdown in C28/I2 chondrocytes.

GO and KEGG analyses revealed that the expression of cell adhesion-related genes and CAMs signaling pathways were significantly decreased after decorin suppression. CAMs can be divided into four families: integrin, cadherin, selectins, and immunoglobulin superfamily [26]. Each of these adhesion molecules recognizes different ligands and performs a different function. In this study, we showed that the integrin (*ITGA4*, *ITGAX*, *ITGB2*, *and ITGB3*) and cadherin families (*CDH3*, *CDH4*, *CDH6*, *CDH8*, *CDH10*, *CDH11*, *CDH12*, *CDH13*, *CDH15*, *CDH23*, *PCDH1*, *PCDH7*, *PCDH9*, *and PCDH10*) (S2 Table) were significantly decreased after decorin suppression. The cadherin family plays a major role in mediating cell-cell interactions, whereas, adhesion between the cell and ECM is mediated by integrins. Han B et al. reported treatment with exogenous decorin increases molecular adhesion between aggrecan-aggrecan molecules and aggrecan-collagen II fibrils in chondrocytes [4]. Articular chondrocytes express various subunits of integrin-α and integrin-β [27]; these integrin bind to a host of cartilage ECM proteins, most notably collagen, fibronectin, laminin, thrombospondin, and vitronectin [28], resulting in the ability to influence various chondrocyte functions, including differentiation, proliferation, and matrix remodeling [29, 30]. Integrin-β mediated chondrocyte-ECM interactions are decreased in osteoarthritic cartilage, which suggests that perturbations of cartilage occur during OA [31, 32]. Additionally, our data also indicates that downregulation of decorin inhibit chondrocytes adhesive ability to fibronectin-coated substrates, multi-adhesive glycoproteins including *FN1* and laminin subunit genes (*LAMA3*, *LAMA4*, *LAMC1*, and *LAMC2*) were significantly decreased in response to decorin knockdown. FN and laminin are multidomain proteins that contain binding sites for integrins, collagen and other ECM proteins [33, 34]. Integrin adhesion complexes directly activate several different signaling cascades, many of the signaling intermediates converge onto the MAP kinase family, regulating the expression of genes that control cell proliferation, migration, and survival [35–37]. Taken together, tightly controlled interactions between adhesion molecules are crucial for tissue development and homeostasis.

Decorin plays an important role in the collagen fibrillogenesis and ECM assembly [2, 38, 39]; these reports are consistent with our analysis. In tissue engineered cartilage, we observed a loss of proteoglycan in the engineered cartilage harvested from shRNA-DCN group. In our RNA-seq data, reduction of decorin expression caused a significant down-regulation of the collagen family (*COL3A1*, *COL4A3*, *COL5A2*, *COL8A1*, *COL12A1*, *COL13A1*, and *COL15A1*) (S2 Table), suggesting the chondrocytes were undergoing a degeneration process due to weakened anabolic activity. These results demonstrated that decorin is one of the important matrix molecules accumulating on the chondrocyte surface. Decorin have affinity for collagen type I, II, III, V, VI, XII, XIV [40–44], and Fibronectin [45]. Decorin knock-out mice exhibit abnormal collagen fibril formation and enhanced collagen degradation [39]. Depletion of decorin causes damage to the collagen network, making the joint structures less suited to withstand physiologic mechanical loading. Nevertheless, no significant difference was detected regarding expression levels of major matrix genes, aggrecan, hyaluronan link protein (Hapln1), Col1a1, and Col2a1. At the same time, the expression level of matrix metalloproteinases (MMP-3, -9, -13), aggrecanase-1(ADAMTS4) and tissue inhibitors of metalloproteinases (TIMPs) remained unchanged. This is consistent with previous studies, which showed that Dcn–/–cartilage exhibits reduced aggrecan content, and loss of decorin reduces the retention of aggrecan, rather than to directly influence the biosynthesis of aggrecan, Hapln1, Col2a1, and Col1a1 in cartilage neo-matrix [4]. In the OA cartilage, increased disruption of collagen fibers accompanies the loss of proteoglycans has been observed, leading to the defective structural integrity of the cartilage [46]. In this study, decorin knockdown active the oxidative phosphorylation signaling pathway, and enhance the expression level of cytochrome c oxidase (COX) and NADH:ubiquinone oxidoreductase subunits, like *COX6B*, *COX7B*, *COX7C*, *COX10*, *NDUFA1*, *NDUFA2*, *NDUFA4*, *NDUFA9*, *NDUFB2*, *NDUFB6*, *NDUFS3*, *NDUFS4*, and *NDUFV2* (S2 Table). Oxidative phosphorylation is a vital part of metabolism, it produces reactive oxygen species (ROS), leads to propagation of free radicals. NADPH oxidase expressed by chondrocytes is the main enzyme responsible for ROS formation, contributing to increased oxidative stress inside the joint and mediating the cartilage collagen degradation, structural destabilization and OA progression [47,48]. Our results indicate that decorin is imperative to maintain the normal metabolic homeostasis.

Accumulating evidence has shown that decorin binding multiple growth factors such as transforming growth factor (TGF-β) [49], TNFα [50], fibroblast growth factor (FGF) [51], and platelet-derived growth factor (PDGF) [52]. Decorin also antagonizes several receptor tyrosine kinases (RTKs), including toll-like receptors (TLRs) [53], insulin-like growth factor receptor (IGFR) [54], ErbB family [55], epidermal growth factor receptor (EGFR) [56], and vascular endothelial growth receptor factor (VEGFR) [57]. In this study, reduction of decorin expression resulted in decreased cell proliferation. GO functional enrichment analysis also revealed that decorin knockdown was significantly associated with altered synthesis of growth factors (*TGFA*, *BMP2*, *BMP5*, *BMP6*, *GDF6*, *GDF9*, *GDF15*, *FGF5*, *FGF12*, *FGF13*, *VEGFC*, *PDGFB*, and *IGF2*) and cell surface receptor (*FGFR2*, *PTGFR*, *PTGER2*, *NGFR*, *TGFBR3*, *ERBB4*, *TLR4*, and *TLR3*) in C28/I2 chondrocytes. The mechanism of modulation of chondrocyte growth and proliferation by decorin could be through interaction with above GFs and RTKs. The FGFs and their receptors play essential roles in skeletal growth and development [58]. *In vivo*, mice conditionally lacking Fgfr2, or harboring a mutation in Fgfr2c exhibited severe growth retardation and decreased bone mineral density due to reduced osteoprogenitor cell proliferation and altered anabolic function of mature osteoblasts [59]. The BMP and GDF are a member of the TGF-β superfamily that are involved in chondrogenesis and chondrocyte proliferation. Bmp2 conditional knockout mice exhibit severe disorganization of chondrocytes within the growth plate region and display profound defects in chondrocyte proliferation and differentiation [60]. GDF15 ablation leads to decreased proliferation and migration in the hippocampus of neonatal and young adult mice [61]. Mutations in IGF-2 or IGF-2R result in growth retardation in both mice and humans [62,63]. PDGF signaling stimulates the chondrocyte proliferation by up-regulating Grb2 and PLCγ1-mediated ERK signaling pathway [64]. Furthermore, decorin tends to bind several different RTKs and induces receptor phosphorylation of specific tyrosines, which in turn leads to the activation of the Ras-MAPK and PI3K/Akt pathway [65]. MAPK and PI3K/Akt pathways are crucial signal transducing systems that are involved in the regulation of important cellular processes, such as cell proliferation and survival. Taken together, as a functional consequence of this multivalent binding abilities, decorin deficiency may affect the bioactivity of chondrocyte proliferation. Furthermore, reduction of decorin expression resulted in increased apoptosis of C28/I2 cells, this finding is inconsistent with previous studies highlighting the ability of decorin to induce apoptosis [66]. Decorin has been reported to trigger apoptosis via caspase-3 activation [67]. In our RNA sequencing data, caspase-3 did not show a significant change by decorin silencing.

In summary, a comprehensive understanding of the molecular mechanisms underlying decorin knockdown was obtained by bioinformatics analyses. RNA-Seq identified differentially expressed genes in C28/I2 cells following decorin down-regulation. Enrichment analysis indicated that diverse cellular processes, including cell adhesion, growth, and metabolism of ECM, were affected by decorin knockdown. Furthermore, *in vitro* study demonstrates that reduction of decorin gene expression in human C28/I2 cells results in a significant decrease in cell proliferation, adhesion and increase in metabolism of ECM. However, the precise mechanisms involved in the interaction between decorin and biological phenotype remain to be elucidated. The addition of recombinant decorin protein partially reversed the effect of shRNA-DCN on gene expression of decorin, IL6, CDH6, CDH11, ICAM3, and BMP5 in C28/I2 chondrocytes (S1 Fig). Further research should be performed to reverse the observed effects in the functional assays for the determination of chondrocyte cell adhesion, proliferation, apoptosis and ECM metabolism by adding to the shRNA-DCN-chondrocytes exogenous decorin. Furthermore, another limitation of this study is that sGAG and collagen content in BMG construct or media were not detected, DMMB, hydroxyproline or immunohistochemical staining for collagen type II should be carried out to estimate the amount of sGAG and newly formed collagen within the BMG construct or supernatants.

## Supporting information

S1 Fig The addition of recombinant decorin protein (20ng/ml) partially reverse the altered gene expression of decorin, IL6, CDH6, CDH11, ICAM3, and BMP5 induced by decorin knockdown in C28/I2 chondrocytes. (TIF)

S1 Raw image (TIF)

S1 Table Differentially expressed genes in C28/I2 chondrocytes following decorin knockdown. (XLSX)

S2 Table List of DEGs related to cell adhesion, growth, ECM metabolism, and oxidative phosphorylation. (DOCX)
