# Supplementary figures and images for: Decorin knockdown affects the gene expression profile of adhesion, growth and extracellular matrix metabolism in C-28/I2 chondrocytes
Source: PLoS One. 2020 Apr 30;15(4):e0232321. doi: 10.1371/journal.pone.0232321 (PMC7192450; doi:10.1371/journal.pone.0232321)

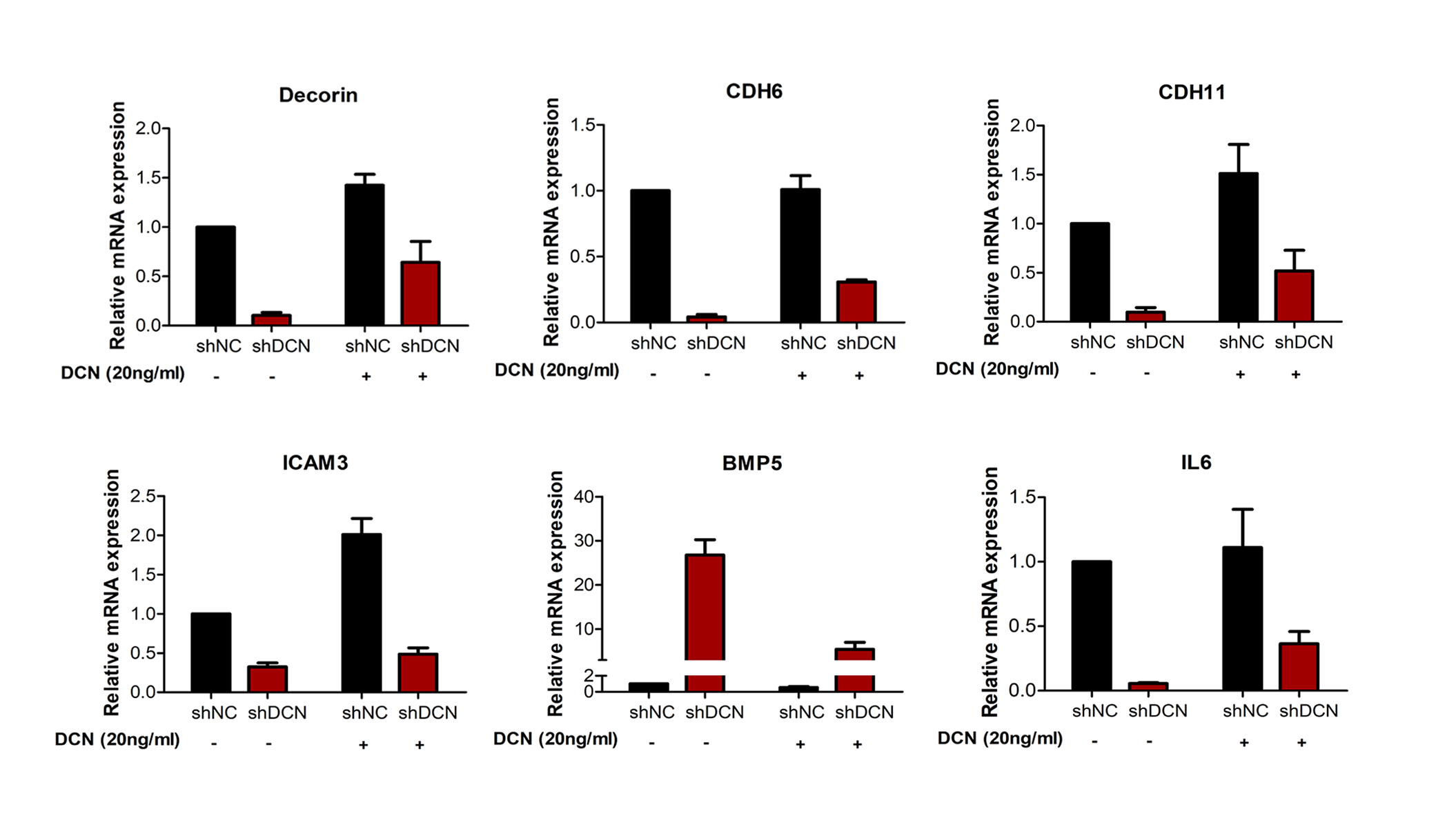

Supplement: S1 Fig — (TIF) [file pone.0232321.s001.tif]

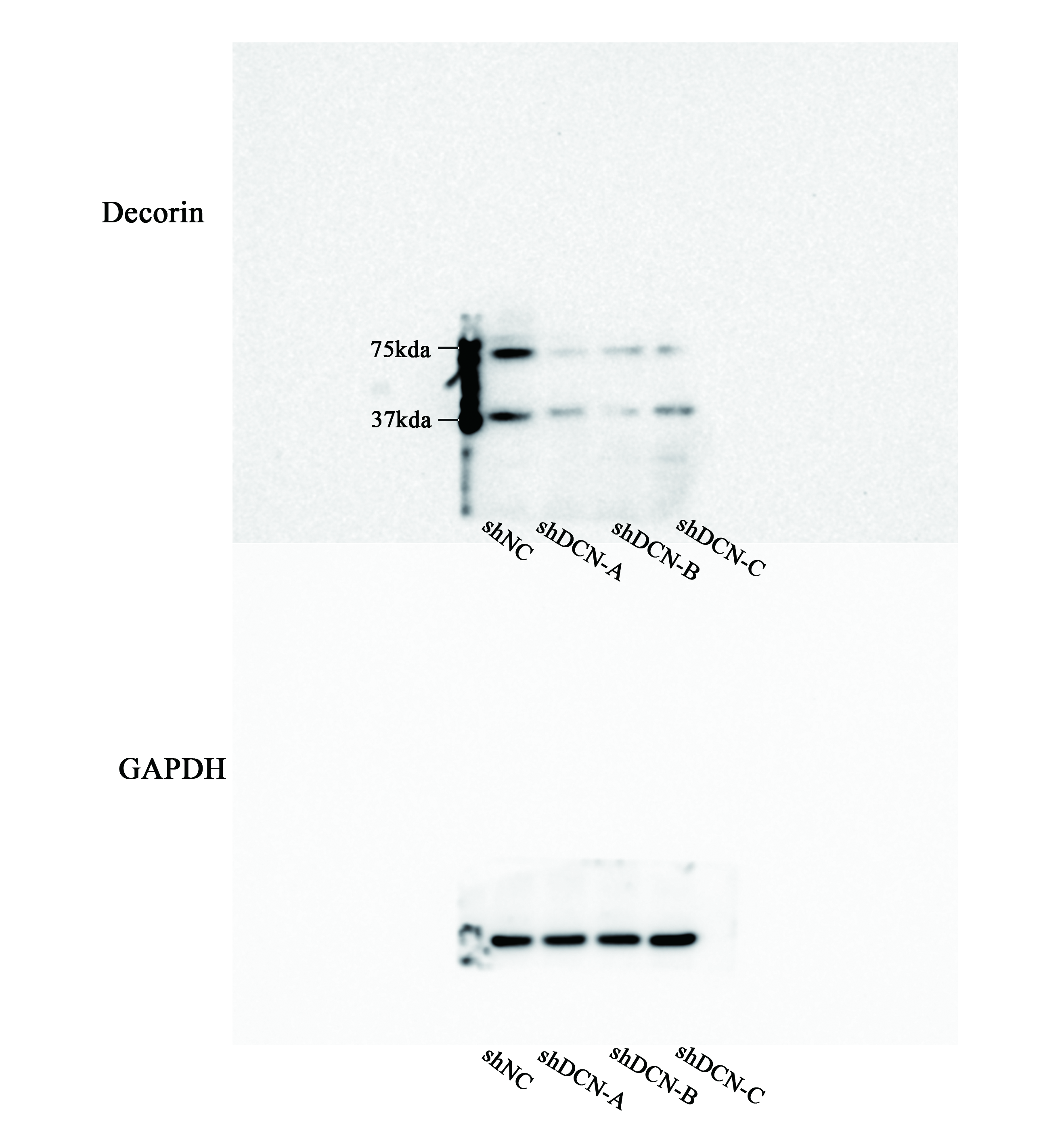

Supplement: S1 Raw image — (TIF) [file pone.0232321.s002.tif]
