# Supplementary material for: Decorin knockdown affects the gene expression profile of adhesion, growth and extracellular matrix metabolism in C-28/I2 chondrocytes
Source: PLoS One. 2020 Apr 30;15(4):e0232321. doi: 10.1371/journal.pone.0232321 (PMC7192450; doi:10.1371/journal.pone.0232321)
Supplement: S2 Table — (DOCX) [file pone.0232321.s004.docx]

**S2 Table.** List of DEGs related to cell adhesion, growth, extracellular matrix metabolism, and oxidative phosphorylation.

| **Gene ID** | **Down-regulated genes** | **Up-regulated genes** |
| --- | --- | --- |
| Cell adhesion molecule (CAMs) | Cadherin family (CDH): CDH3, CDH4, CDH6, CDH8, CDH10, CDH11, CDH12, CDH13, CDH15, CDH23, PCDH1, PCDH7, PCDH9, PCDH10;  Integrin family (ITG): ITGA4, ITGAX, ITGB2, ITGB3;  Others: ICAM3, MCAM, L1CAM, NCAM2, NRCAM | ITGA10, LAMA1, EPCAM |
|  | laminin family (LAM): LAMA3, LAMC2, LAMA4, LAMC1; |  |
| Collagen (Col) family | COL3A1, COL4A3, COL5A2, COL8A1, COL12A1, COL13A1, COL15A1, | COL20A1, COL22A1 |
| Growth factors (GFs) | IGF2, TGFA, BMP2, VEGFC, PDGFB, FGF5, FGF12, FGF13, GDF15, | BMP5, BMP6, GDF6, GDF9 |
| Receptor tyrosine kinases (RTKs) | ERBB4, NGFR, PTGFR, TLR4, TLR3 | FGFR2, TGFBR3, PTGER2, |
| cytochrome c oxidase subunit (COX) | COX6B2 | COX6B1, COX7B, COX7C, COX10, COX16 |
| NADH:ubiquinone oxidoreductase | N/A | NDUFA1, NDUFA2, NDUFA4, NDUFA9, NDUFB2, NDUFB6, NDUFS3, NDUFS4, NDUFV2 |

ICAM3, intercellular adhesion molecule 3; MCAM, melanoma cell adhesion molecule; L1CAM, L1 cell adhesion molecule; NCAM2, neural cell adhesion molecule 2; NRCAM, neuronal cell adhesion molecule; EPCAM, epithelial cell adhesion molecule; IGF2, insulin like growth factor 2; BMP2, bone morphogenetic protein 5; VEGFC, vascular endothelial growth factor C; PDGFB, platelet derived growth factor subunit B; FGF5, fibroblast growth factor 5; GDF15, growth differentiation factor 15; ERBB4, erb-b2 receptor tyrosine kinase 4; NGFR, nerve growth factor receptor; PTGFR, prostaglandin F receptor; TLRs: toll-like receptors; EGFR: epidermal growth factor receptor; VEGFR: vascular endothelial growth factor receptor; IGF-1R: insulin-like growth factor receptor-1.
